# EASIX Score as an Independent Prognostic Marker in De Novo Metastatic Pancreatic Cancer

**DOI:** 10.3390/medicina62030562

**Published:** 2026-03-18

**Authors:** İlkay Çıtakkul, Yasemin Bakkal Temi, Zehra Aytin, Ece Baydar, Umut Kefeli, Devrim Çabuk, Kazım Uygun

**Affiliations:** Department of Internal Medicine and Medical Oncology, Kocaeli University, 41380 Kocaeli, Turkey; yasemin.temi@kocaeli.edu.tr (Y.B.T.); dr.zehraonesu@gmail.com (Z.A.); ecebaydar@gmail.com (E.B.); ukefeli@yahoo.com (U.K.); devrimcabuk@yahoo.com (D.Ç.); kzuygun@hotmail.com (K.U.)

**Keywords:** pancreatic cancer, EASIX score, overall survival, endothelial dysfunction, risk stratification

## Abstract

*Background and Objectives*: Metastatic pancreatic ductal adenocarcinoma carries a dismal prognosis and there is an unmet need for simple, widely available prognostic biomarkers to guide risk stratification and treatment planning. This study aimed to evaluate whether baseline EASIX score, calculated from routine laboratory parameters (LDH, creatinine, platelet count), predicts overall survival (OS) in patients with de novo metastatic pancreatic cancer. *Materials and Methods*: We performed a retrospective cohort study at a single tertiary center (Medical Oncology Department, Kocaeli University Faculty of Medicine) including 332 patients diagnosed with de novo metastatic pancreatic ductal adenocarcinoma between January 2019 and October 2025. Baseline EASIX was calculated using LDH, creatinine, and platelet count. Statistical analyses included ROC analysis with Youden index to determine exploratory cut-off, Kaplan–Meier survival estimation with log-rank test, and univariate and multivariate Cox proportional hazards regression to identify independent prognostic factors. The primary endpoint was OS, defined as time from initiation of first-line therapy to death from any cause. *Results*: A total of 332 patients were included. Median OS was 8.0 months overall. Patients with high EASIX (>2.505) had significantly shorter median OS compared with low EASIX patients (8.4 vs. 27.6 months, *p* < 0.001). ROC analysis was considered exploratory for overall survival; therefore, an additional fixed-time (12-month mortality) ROC analysis was performed to provide a discrimination estimate accounting for censoring. The AUC for EASIX was 0.887 (95% CI: 0.816–0.958), and the exploratory cut-off determined was 2.505 (sensitivity 96.95%, specificity 72.97%). In multivariate Cox regression, high EASIX remained an independent predictor of worse OS (HR 4.124; 95% CI: 2.011–8.457; *p* < 0.001) after adjustment for relevant covariates. *Conclusions*: Baseline EASIX score is an independent prognostic marker for overall survival in de novo metastatic pancreatic cancer, based on routine laboratory tests, and may facilitate exploratory risk stratification. Prospective validation in independent cohorts is warranted before clinical implementation.

## 1. Introduction

Pancreatic cancer is recognized as one of the most aggressive and lethal malignancies, characterized by a subtle onset, delayed clinical manifestation, and resistance to standard therapeutic interventions. Globally, it is the seventh leading cause of cancer-related mortality, and although it constitutes only approximately 3% of all cancer cases, it accounts for a disproportionately high number of cancer-related deaths [1]. The prognosis is particularly dire for patients diagnosed at the metastatic stage, where the 5-year survival rate remains below 10%, and the median overall survival (OS) seldom surpasses one year, even with combination chemotherapy [2]. In light of these challenges, there is an urgent need to identify reliable, accessible, and cost-effective prognostic markers that can inform therapeutic decisions and facilitate patient risk stratification.

While tumor staging and performance status are traditionally employed to predict outcomes, these parameters may not fully encapsulate the biological heterogeneity of pancreatic cancer. Increasing attention has been directed toward biomarkers derived from routine blood tests, which may provide insights into the tumor microenvironment, systemic inflammation, and host response to the disease [3]. One such marker is the Endothelial Activation and Stress Index (EASIX)—a composite index calculated from lactate dehydrogenase (LDH), serum creatinine, and platelet count. Initially proposed to predict graft-versus-host disease-related complications in allogeneic stem cell transplantation, EASIX has subsequently been validated in several hematologic malignancies [4,5,6,7,8].

Recent research has elucidated the prognostic significance of the EASIX score in diverse clinical settings. EASIX has also demonstrated relevance in solid tumors, including small cell lung cancer, urothelial cancer and metastatic pancreatic cancer [9,10,11]. Furthermore, its predictive utility has been investigated in chronic inflammatory and cardiovascular diseases, such as asthma, COVID-19, heart failure, and coronary artery disease, where elevated EASIX scores have been correlated with an increased risk of morbidity and mortality [12,13,14,15,16,17]. In the context of metastatic pancreatic cancer, the prognostic significance of EASIX is currently supported by evidence from only a single published study [10]. Given the systemic and biologically aggressive nature of this malignancy, EASIX may serve as an informative marker to support risk-adapted treatment approaches.

EASIX is posited to reflect endothelial dysfunction and systemic stress, both of which are increasingly acknowledged as central features in cancer progression, metastasis, and resistance to treatment. Elevated LDH levels indicate hypoxic tumor metabolism and aggressive growth; elevated creatinine may signal systemic stress or organ dysfunction; and thrombocytosis or thrombocytopenia can denote inflammatory activation or bone marrow suppression. Collectively, these parameters provide insight into both tumor biology and host response, offering a dynamic assessment of disease aggressiveness [3].

Moreover, the practicality of EASIX—based on routine, inexpensive laboratory parameters—renders it particularly attractive for resource-limited settings and routine clinical use. As a non-invasive and reproducible index, EASIX could be integrated into standard baseline assessments and used to complement existing clinical scoring systems.

In this study, we aimed to investigate the prognostic value of the EASIX score in patients with de novo metastatic pancreatic adenocarcinoma receiving first-line systemic chemotherapy. We sought to determine whether EASIX, measured prior to treatment initiation, could independently predict OS, and whether it could serve as a practical tool for early risk stratification and individualized clinical management.

## 2. Materials and Methods

### 2.1. Study Design and Patient Population

This retrospective cohort study was conducted within the Medical Oncology Department at Kocaeli University Faculty of Medicine. The medical records of patients diagnosed with de novo metastatic pancreatic ductal adenocarcinoma between January 2019 and October 2025 were examined. Inclusion criteria required histopathological confirmation via biopsy from either the primary tumor or a metastatic site, alongside radiological verification of metastatic disease through appropriate cross-sectional imaging modalities, such as computed tomography (CT), magnetic resonance imaging (MRI), or positron emission tomography (PET-CT). Patients with non-metastatic disease or those who developed metastasis following initial treatment were excluded. All patients who underwent at least one cycle of first-line chemotherapy and possessed complete baseline laboratory parameters were included in the analysis, irrespective of treatment duration. Patients were required to have available baseline laboratory data prior to treatment initiation, including serum lactate dehydrogenase (LDH), creatinine, platelet count, and other routinely recorded laboratory variables used in exploratory survival analyses (e.g., body mass index (BMI), C-reactive protein (CRP), and serum albumin). Additional exclusion criteria included prior chemotherapy for metastatic disease, the presence of concurrent malignancies, or incomplete clinical or laboratory documentation.

### 2.2. Data Collection

Demographic characteristics, including age and sex, clinical features such as Eastern Cooperative Oncology Group (ECOG) performance status and sites of metastasis, laboratory parameters including hemoglobin, neutrophil, lymphocyte, lactate dehydrogenase (LDH), creatinine, platelet count, carcinoembryonic antigen (CEA), and carbohydrate antigen 19-9 (CA 19-9), and treatment-related data, specifically the type of first-line chemotherapy, were extracted from electronic medical records. Inflammatory markers, including the neutrophil-to-lymphocyte ratio (NLR) and the systemic immune-inflammation index (SII, calculated as platelet count multiplied by neutrophil count divided by lymphocyte count), were also derived. Survival status was verified through hospital records and national death registries. The primary endpoint was OS, defined as the duration from the initiation of first-line therapy to death from any cause. The EASIX score was determined using the standard formula: EASIX = (LDH [U/L] × serum creatinine [mg/dL])/platelet count [10^9^/L]. In our institutional laboratory reporting system, platelet counts are recorded as ×10^3^/µL, which is numerically equivalent to 10^9^/L; thus, no additional unit conversion was necessary prior to calculation. No logarithmic transformation, normalization, or scaling was applied at any stage, and all analyses were conducted using raw EASIX values. Consequently, the cut-off value reported in this study corresponds to the original EASIX scale and can be directly applied using the formula and unit conventions specified above.

In the survival analyses, additional baseline variables included BMI, CRP, and serum albumin. All laboratory parameters were derived from routine blood tests conducted prior to the commencement of first-line chemotherapy, with the values closest to the initiation of treatment utilized for analysis. BMI was calculated as weight (kg) divided by height squared (m^2^), based on measurements recorded at the start of treatment. CRP was measured in mg/L, and serum albumin was measured in g/dL, in accordance with institutional laboratory standards.

### 2.3. Statistical Analysis

Descriptive statistics were employed to summarize baseline characteristics. Categorical variables were compared using the Chi-square or Fisher’s exact test, while continuous variables were analyzed using the Mann–Whitney U test. Receiver Operating Characteristic (ROC) analysis was conducted to explore the discriminatory performance of EASIX. Because overall survival represents a censored time-to-event outcome, the ROC analysis for OS was interpreted as exploratory. To provide a clinically interpretable discrimination measure while accounting for censoring, an additional ROC analysis was performed for 12-month mortality as a fixed time horizon. The area under the curve (AUC), sensitivity, specificity, and exploratory cut-off values based on Youden’s index were reported.

In parallel, the prognostic impact of EASIX was evaluated using Cox proportional hazards regression models. EASIX was examined both as a dichotomized variable based on the exploratory cut-off and as a continuous variable to assess its association with overall survival independent of categorization. These complementary approaches were used to ensure a robust evaluation of discrimination and prognostic association. Survival curves were estimated using the Kaplan–Meier method and compared using the log-rank test. Univariate and multivariable Cox proportional hazards regression analyses were performed to identify factors associated with OS. Multivariable models included clinically relevant covariates selected a priori (age, tumor markers, first-line treatment, and major metastatic sites). Continuous variables were entered using their raw values without transformation. The proportional hazards assumption was assessed graphically and was not violated. In the primary multivariable models, EASIX was entered as a dichotomized variable based on the exploratory cut-off, while analyses treating EASIX as a continuous variable were performed in univariate Cox models. Consequently, although NLR and SII were initially considered as potential prognostic biomarkers, they were excluded from the multivariate analysis due to their lack of statistical significance in the univariate testing. Hazard ratios (HR) and 95% confidence intervals (CI) were reported. Statistical significance was determined at *p* < 0.05. All statistical analyses were performed using IBM SPSS Statistics for Windows version 29.0 (IBM Corp., Armonk, NY, USA).

## 3. Results

A cohort of 332 patients diagnosed with metastatic pancreatic cancer was included in this study. The median age of the participants was 65 years (IQR: 58–71), with 32.8% of the patients being 70 years or older. The study population comprised 59.5% males. In terms of performance status, 40.1% of the patients had an Eastern Cooperative Oncology Group (ECOG) score of 0, 50.0% had a score of 1, and 9.9% had a score of 2. For first-line chemotherapy, 76.8% of the patients received FOLFIRINOX, while 23.2% were administered gemcitabine-based regimens. Liver metastases were present in 74.7% of patients, lymph node metastases in 51.8%, bone metastases in 42.8%, and peritoneal/implant metastases in 23.8%. A summary of the clinical and laboratory characteristics is provided in Table 1.

**Figure 1:**
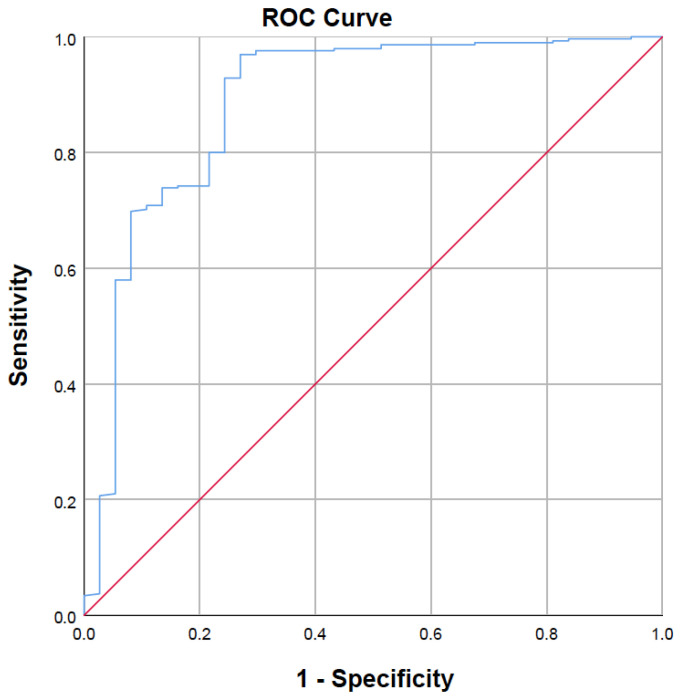
Receiver Operating Characteristic (ROC) curve demonstrating the diagnostic performance of the EASIX score in predicting survival. The area under the curve (AUC) was 0.887 (95% CI: 0.816–0.958, *p* < 0.001), indicating high discriminative ability. The exploratory cut-off value determined by Youden’s Index was 2.505, yielding a sensitivity of 96.95% and specificity of 72.97%. The red curve represents the ROC plot of the EASIX score, while the blue diagonal line indicates the reference line (no-discrimination).

The receiver operating characteristic (ROC) curve analysis was conducted to assess the discriminative capability of the EASIX score in predicting survival outcomes. The area under the curve (AUC) was determined to be 0.887 (95% CI: 0.816–0.958, *p* < 0.001), signifying a high degree of accuracy. Utilizing Youden’s Index, the exploratory cut-off value was established at 2.505, yielding a sensitivity of 96.95% and a specificity of 72.97% (Figure 1).

ROC curve analyses were performed to assess the predictive efficacy of the neutrophil-to-lymphocyte ratio (NLR) and the systemic immune-inflammation index (SII) concerning OS. For NLR, the area under the curve (AUC) was calculated to be 0.496 (95% CI: 0.382–0.610, *p* = 0.944). Utilizing Youden’s Index, the exploratory cut-off value was determined to be 1.825, resulting in a sensitivity of 88.47%, specificity of 24.32%, positive predictive value of 90.31%, and negative predictive value of 20.93%. Similarly, the AUC for SII was found to be 0.486 (95% CI: 0.372–0.600, *p* = 0.805). The exploratory cut-off value, ascertained through Youden’s Index, was 329.845, with a sensitivity of 92.54%, specificity of 18.92%, positive predictive value of 90.10%, and negative predictive value of 24.14%. To account for censoring in overall survival analyses, an additional ROC analysis was performed for 12-month mortality. The AUC of EASIX for predicting 12-month mortality was 0.850 (95% CI 0.776–0.923, *p* < 0.001). Using Youden’s index, the exploratory cut-off yielded a sensitivity of 72.97% and a specificity of 96.95%.

Patients were stratified into low and high EASIX score groups and compared in terms of clinical and laboratory characteristics. There were no statistically significant differences between the groups regarding age, gender, ECOG performance status, or type of first-line chemotherapy (all *p* > 0.05). Similarly, neutrophil-to-lymphocyte ratio (NLR), systemic immune-inflammation index (SII), neutrophil count, lymphocyte count, hemoglobin, platelet count, creatinine, CEA, and CA 19-9 levels did not differ significantly between the groups.

However, lactate dehydrogenase (LDH) levels were significantly higher in the high EASIX group compared to the low EASIX group (1021 vs. 427 U/L, *p* < 0.001). No statistically significant differences were observed in the distribution of metastatic sites, including liver, lymph node, bone, and peritoneal/implant metastases. Further details are presented in Table 2.

**Figure 2:**
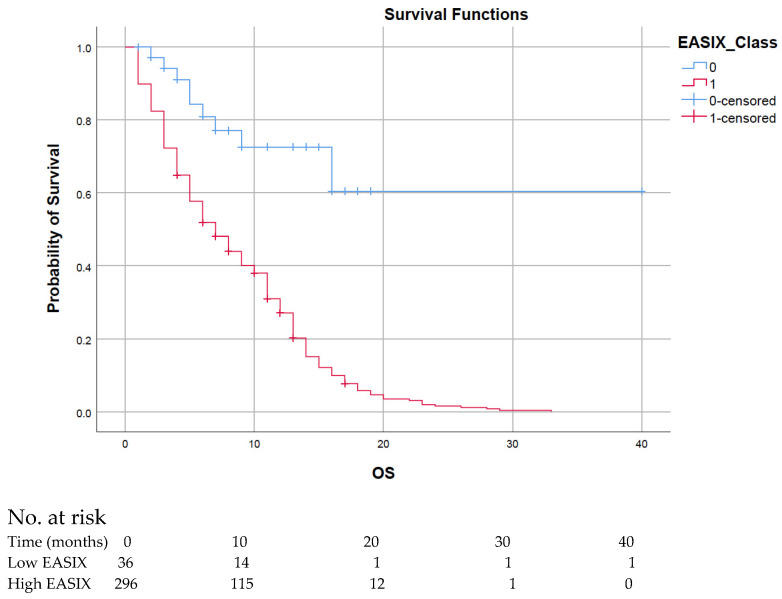
Kaplan–Meier OS Curve According to EASIX Score. Patients with high EASIX scores (red line) exhibited a significantly shorter median OS (month) compared to those with low EASIX scores (blue line) (*p* < 0.001). Numbers at risk are shown below the Kaplan–Meier curves.

The median follow-up time was 40 months. Kaplan–Meier analysis was employed to assess OS in relation to EASIX score groups. The median OS for the entire cohort was 8.0 months (95% CI: 6.7–9.3). Among patients with high EASIX scores, the median OS was 8.4 months (95% CI: 7.7–9.1), whereas it was significantly extended to 27.6 months (95% CI: 20.3–34.8) in the low EASIX group. The log-rank test revealed a statistically significant difference between the groups (*p* < 0.001), indicating a poorer prognosis for patients with high EASIX scores. During the follow-up period, a total of 295 deaths were recorded. Within the low EASIX group, there were 9 deaths and 27 censored observations. In contrast, the high EASIX group experienced 286 deaths and 10 censored observations. For a detailed analysis of OS by EASIX score, refer to Figure 2.

In the univariate Cox regression analysis, several factors were identified as significant prognostic indicators for OS. These included age (continuous) (HR: 1.018, 95% CI: 1.006–1.031, *p* = 0.003), CA 19-9 levels (HR: 1.116, 95% CI: 1.060–1.174, *p* < 0.001), CEA levels (HR: 1.253, 95% CI: 1.164–1.349, *p* < 0.001), liver metastasis (HR: 1.524, 95% CI: 1.162–1.998, *p* = 0.002), intra-abdominal lymph node metastasis (HR: 1.674, 95% CI: 1.328–2.110, *p* < 0.001), and bone metastasis (HR: 1.926, 95% CI: 1.523–2.437, *p* < 0.001). Upon evaluation using the EASIX classification, patients categorized within the high EASIX group exhibited a significantly elevated risk of mortality compared to those in the low EASIX group (HR: 4.709, 95% CI: 2.422–9.155, *p* < 0.001), thereby indicating substantially poorer OS. Detailed results are presented in Table 3.

In the multivariate Cox regression analysis, elevated EASIX scores remained a strong independent prognostic factor for OS. Patients in the high EASIX group had significantly worse OS compared with those in the low EASIX group (HR: 4.310, 95% CI: 2.103–8.837, *p* < 0.001). Among tumor markers, CEA was independently associated with OS (HR: 1.194, 95% CI: 1.093–1.304, *p* < 0.001). In terms of metastatic burden, intra-abdominal lymph node metastasis (HR: 1.374, 95% CI: 1.047–1.803, *p* = 0.022) and bone metastasis (HR: 1.801, 95% CI: 1.366–2.376, *p* < 0.001) were independently associated with worse survival. Age (continuous), CA 19-9, liver metastasis, CRP, albumin, and first-line chemotherapy regimen were not independently associated with OS in the multivariate model. Detailed results are presented in Table 4.

## 4. Discussion

In this study, we examined the prognostic significance of the EASIX score in patients with metastatic pancreatic cancer undergoing first-line chemotherapy. Our results indicated that a high EASIX score independently predicted significantly poorer OS, even after adjusting for clinical and pathological variables. Patients in the high EASIX group exhibited notably shorter survival compared to those in the low EASIX group. These findings endorse the use of EASIX as a prognostic tool in metastatic pancreatic cancer and are consistent with the expanding body of evidence from both solid tumors and hematologic malignancies [4,5,6,9].

The prognostic relevance of the EASIX score likely reflects the combined biological information captured by its components, including LDH, creatinine, and platelet count. Rather than representing independent findings, these parameters together provide a composite measure of systemic stress, endothelial dysfunction, and tumor–host interaction. Our findings align with those of Cavdar et al., who demonstrated that an elevated EASIX score was associated with significantly worse overall survival in metastatic pancreatic cancer [10]. In contrast to the approach taken by Cavdar et al., which utilized a threshold based on lymphoma data, our study established an EASIX cutoff specifically designed for patients with metastatic pancreatic cancer. This tumor-specific calibration enhances the prognostic accuracy and clinical applicability of the score within this distinct context.

In our study, the EASIX score exhibited significant prognostic discrimination, with a median OS of 27.6 months in the low-risk group, compared to 8.4 months in the high-risk group. The median survival of the overall cohort, at 8.0 months, is consistent with previously reported outcomes in metastatic pancreatic cancer, thereby reinforcing the validity of our findings. The significantly extended survival observed in the low EASIX group may be attributed to the preservation of endothelial integrity and a reduced systemic inflammatory burden, both of which are biologically associated with more favorable tumor behavior [18,19,20]. Furthermore, unmeasured confounding factors, such as molecular subtype or superior functional reserve, may also contribute to this outcome [21]. These results further substantiate the clinical utility and prognostic efficacy of the EASIX score for risk stratification.

In multivariate Cox regression analysis, a high EASIX score was identified as an independent predictor of poorer OS (HR: 4.094, 95% CI: 1.993–8.411, *p* < 0.001). This association persisted even after adjusting for critical clinical variables, including age, CEA levels, and the presence of liver, intra-abdominal lymph node, and bone metastases. Although bone metastases are relatively uncommon in pancreatic cancer, their presence is associated with a significantly worse prognosis and reflects a more aggressive tumor phenotype [22]. These findings highlight the potential utility of EASIX as a robust prognostic marker that extends beyond traditional clinicopathological factors.

In our cohort, patients were administered two distinct first-line chemotherapy regimens—FOLFIRINOX and gemcitabine-based protocols. However, no statistically significant difference in OS was observed between these groups (*p* = 0.884). Furthermore, univariate Cox regression analysis did not identify the chemotherapy regimen as a significant predictor of OS. These findings indicate that the prognostic relevance of the EASIX score is independent of the initial chemotherapy strategy. Notably, Cavdar et al. assessed the prognostic value of the EASIX score in a more homogeneous population, exclusively comprising patients treated with FOLFIRINOX [10]. In contrast, the present study broadens the applicability of EASIX by including patients treated with both FOLFIRINOX and gemcitabine-based regimens.

Beyond pancreatic cancer, the prognostic significance of EASIX has been demonstrated in other malignancies. For instance, in patients with diffuse large B-cell lymphoma (DLBCL), EASIX has been shown to predict one-year OS and progression-free survival (PFS), independent of classical clinical prognostic factors [7,8]. Similarly, studies in small cell lung cancer (SCLC) have indicated that patients with high EASIX scores have significantly shorter PFS and OS, supporting the index’s prognostic utility in various solid tumors [9]. These findings suggest that EASIX is not confined to a single cancer type and may serve as a broader oncologic biomarker.

The prognostic significance of EASIX has been investigated in non-oncologic conditions as well. Specifically, in systemic disorders where endothelial damage is pivotal, such as diabetes and prediabetes, elevated EASIX scores have been correlated with all-cause mortality. Similarly, in chronic systemic diseases like liver disease, EASIX has been associated with OS. These findings suggest that EASIX may function not only as a cancer biomarker but also as an indicator of endothelial injury in broader clinical contexts [16,23,24,25].

The prognostic relevance of EASIX likely reflects the combined biological information captured by its components. Rather than representing independent findings, these parameters together provide a composite measure of systemic stress and tumor–host interaction. A reduced platelet count may indicate bone marrow suppression or systemic inflammation. Abnormal creatinine levels reflect renal function and systemic stress. The integration of these parameters into a single index provides a comprehensive reflection of both tumor biology and host response [17,26].

In aggressive tumors such as pancreatic cancer, there is a distinct need for readily accessible biomarkers that can predict treatment response and survival. While current prognostic models often rely on performance status and staging, they may not fully capture biological heterogeneity. Our study demonstrates that EASIX may help address this gap by offering a practical and cost-effective prognostic tool. We posit that EASIX, when used alongside existing clinical scoring systems, could enable more accurate identification of high-risk patients and contribute to individualized treatment planning.

When compared with other widely used prognostic scores in oncology, EASIX offers several advantages. For instance, the International Prognostic Index (IPI) used in lymphoma includes age, LDH, and performance status among other parameters. However, such systems are typically disease-specific and may not be applicable across different cancers. Furthermore, its consistent prognostic performance across tumor types suggests a common role for endothelial dysfunction in cancer progression [3,21].

Our findings indicate several significant clinical applications for EASIX in the management of metastatic pancreatic cancer. This score may function as a practical tool for early risk stratification at diagnosis or prior to the initiation of treatment, enabling clinicians to identify high-risk patients who might benefit from more intensive therapy or closer surveillance. Furthermore, EASIX may assist in refining therapeutic decision-making and follow-up strategies. From a research perspective, incorporating EASIX as a stratification variable in future clinical trials may enhance baseline risk balance. As our understanding of the biological mechanisms underlying EASIX progresses, this index may also contribute to the development of novel therapies targeting endothelial dysfunction and systemic inflammation.

Several limitations of this study warrant acknowledgment. Firstly, the retrospective and single-center design may introduce a risk of selection bias and unmeasured confounding, despite the application of multivariable adjustments. Secondly, the smaller number of patients in the low-EASIX subgroup compared to the high-EASIX group may have influenced the observed magnitude of survival differences between these groups; thus, these findings should be interpreted within the context of the study design. Thirdly, the EASIX cut-off value was empirically derived using ROC analysis and may vary across different populations; therefore, external validation in independent cohorts is necessary before considering routine clinical implementation. Additionally, although ROC analysis for 12-month mortality was performed to address censoring in survival data, time-dependent ROC and external validation were not conducted and should be explored in future research. Finally, treatment heterogeneity and potential differences in baseline biological characteristics, which may not be fully captured by routine clinical variables, could have influenced survival outcomes. In particular, the median OS observed in the low-EASIX subgroup should be interpreted with caution, as this group included a relatively small number of patients and events with a high proportion of censored observations, which may lead to an overestimation of Kaplan–Meier median survival.

## 5. Conclusions

The EASIX score represents a readily applicable, non-invasive biomarker with potential utility for prognostic stratification in metastatic pancreatic cancer. Its incorporation into clinical evaluation may facilitate risk stratification and personalized management; however, these findings should be regarded as exploratory. Prospective, multicenter studies and external validation in independent cohorts are necessary prior to routine clinical implementation. Additionally, further research should explore dynamic changes in EASIX during treatment.

## Figures and Tables

**Table 1:** Baseline Characteristics of the Study Population (Total *N* = 332).

Variable	Median (IQR)	*n* (%) (*N* = 332)
Age (year)	65.0 (58.0–71.0)	
≥70 years		109 (32.8%)
<70 years		223 (67.2%)
Gender		
Male		198 (59.5%)
Female		134 (40.5%)
ECOG Score		
0		133 (40.1%)
1		166 (50.0%)
2		33 (9.9%)
BMI (kg/m^2^)	24.3 (21.8–27.3)	
Albumin (g/dL)	3.97 (3.60–4.40)	
CRP (mg/L)	19.0 (5.9–63.4)	
First-Line Chemotherapy		
FOLFIRINOX		255 (76.8%)
Gemcitabine-based		77 (23.2%)
Survival Status		
Alive		37 (11.1%)
Dead		295 (88.9%)
Neutrophil-to-Lymphocyte Ratio	3.36 (2.25–5.06)	
Systemic Immune-Inflammation Index (SII)	885.72 (548.74–1141.67)	
Neutrophil Count (×10^3^/µL)	5606 (4092–8050)	
Lymphocyte Count (×10^3^/µL)	1724 (1161–2190)	
Hemoglobin (g/dL)	12.6 (11.1–13.7)	
Platelet Count (×10^3^/µL)	258 (199–325)	
Creatinine (mg/dL)	0.79 (0.6–0.96)	
Lactate Dehydrogenase (U/L)	931 (653–1349)	
CEA (ng/mL)	6.2 (2.7–25.6)	
CA 19-9 (U/mL)	1000 (153–5715)	
EASIX Score	2.99 (2.72–3.24)	
Liver Metastasis		
Present		248 (74.7%)
Absent		84 (25.3%)
Lymph Node Metastasis		
Present		172 (51.8%)
Absent		160 (48.2%)
Bone Metastasis		
Present		142 (42.8%)
Absent		190 (57.2%)
Peritoneal/Implant Metastasis		
Present		79 (23.8%)
Absent		253 (76.2%)

Abbreviations: ECOG, Eastern Cooperative Oncology Group; BMI, body mass index; CRP, C-reactive protein; FOLFIRINOX, 5-fluorouracil, leucovorin, irinotecan, and oxaliplatin; NLR, neutrophil-to-lymphocyte ratio; SII, systemic immune-inflammation index; LDH, lactate dehydrogenase; CEA, carcinoembryonic antigen; CA 19-9, carbohydrate antigen 19-9; EASIX, endothelial activation and stress index; IQR, interquartile range; µL, microliter; g/dL, grams per deciliter; mg/dL, milligrams per deciliter; U/L, units per liter.

**Table 2:** Comparison of Baseline Characteristics According to EASIX Score Groups. (Total *N* = 332).

Variable	Low EASIX (*n* = 36, %)	High EASIX (*n* = 296, %)	*p*-Value
Age (year)			
70≥	11 (30.6%)	98 (33.1%)	0.758
70<	25 (69.4%)	198 (66.9%)	
Gender			
Male	20 (55.6%)	178 (60.1%)	0.597
Female	16 (44.4%)	118 (39.9%)	
ECOG Score			
0	12 (33.3%)	121 (40.9%)	0.683
1	20 (55.6%)	146 (49.3%)	
2	4 (11.1%)	29 (9.8%)	
First-Line Chemotherapy			
FOLFIRINOX	28 (77.8%)	227 (76.7%)	0.884
Gemcitabine-based	8 (22.2%)	69 (23.3%)	
Neutrophil-to-Lymphocyte Ratio median (IQR)	3.07 (2.09–5.09)	3.43 (2.24–5.08)	0.357
Systemic Immune-Inflammation Index (SII) median (IQR)	845.72 (513.82–1679.83)	942.02 (546.07–1441.52)	0.379
Neutrophil Count (×10^3^/µL) median (IQR)	5570 (3941–8290)	5580 (4200–7790)	0.642
Lymphocyte Count (×10^3^/µL) median (IQR)	1760 (1300–2060)	1710 (1134–2160)	0.446
Hemoglobin (g/dL) median (IQR)	12.5 (11.2–13.3)	12.6 (11.3–13.8)	0.619
CEA (ng/mL) median (IQR)	4.45 (2.22–8.56)	7.40 (2.70–28)	0.105
CA 19-9 (U/mL) median (IQR)	460 (161–3727)	961 (151–6003)	0.394
Liver Metastasis			0.114
Present	23 (63.9%)	225 (76.0%)	
Absent	13 (36.1%)	71 (24.0%)	
Lymph Node Metastasis			0.100
Present	14 (38.9%)	158 (53.4%)	
Absent	22 (61.1%)	138 (46.6%)	
Bone Metastasis			0.887
Present	15 (41.7%)	127 (42.9%)	
Absent	21 (58.3%)	169 (57.1%)	
Peritoneal/Implant Metastasis			0.200
Present	5 (13.9%)	69 (23.3%)	
Absent	31 (86.1%)	227 (76.7%)	

Abbreviations: IQR: interquartile range; SII: systemic immune-inflammation index; CEA: carcinoembryonic antigen; CA 19-9: carbohydrate antigen 19-9; EASIX: endothelial activation and stress index; ECOG: Eastern Cooperative Oncology Group; FOLFIRINOX: a multi-agent chemotherapy regimen consisting of 5-fluorouracil, leucovorin, irinotecan, and oxaliplatin.

**Table 3:** Univariate Cox Regression Analysis for OS.

Variable	HR (95% CI)	*p*-Value
Age (continuous)	1.018 (1.006–1.031)	0.003
Gender (Male vs. Female)	1.007 (0.797–1.271)	0.954
BMI	1.014 (0.990–1.040)	0.257
First-Line Chemotherapy (FX vs. Gem)	1.010 (0.770–1.324)	0.944
Neutrophil count	0.935 (0.838–1.042)	0.225
Lymphocyte count	0.917 (0.809–1.038)	0.172
CRP	1.005 (0.927–1.088)	0.908
Albumin	1.140 (0.952–1.366)	0.153
NLR Group	1.283 (0.897–1.837)	0.173
SII Group	1.400 (0.905–2.164)	0.130
EASIX Group (High vs. Low)	4.709 (2.422–9.155)	<0.001
EASIX Group (continuous)	1.784 (1.375–2.314)	<0.001
CA 19-9	1.116 (1.060–1.174)	<0.001
CEA	1.253 (1.164–1.349)	<0.001
Liver Metastasis	1.524 (1.162–1.998)	0.002
Intra-abdominal LN Metastasis	1.674 (1.328–2.110)	<0.001
Peritoneal Metastasis	1.139 (0.873–1.487)	0.337
Intra-abdominal Implant Metastasis	1.251 (0.956–1.637)	0.103
Bone Metastasis	1.926 (1.523–2.437)	<0.001

Abbreviations: OS, overall survival; HR, hazard ratio; CI, confidence interval; BMI, body mass index; ECOG, Eastern Cooperative Oncology Group; CRP, C-reactive protein; NLR, neutrophil-to-lymphocyte ratio; SII, systemic immune-inflammation index; EASIX, endothelial activation and stress index; CA 19-9, carbohydrate antigen 19-9; CEA, carcinoembryonic antigen; LN, lymph node; FX, FOLFIRINOX (5-fluorouracil, leucovorin, irinotecan, oxaliplatin); Gem, gemcitabine-based regimen.

**Table 4:** Multivariate Cox Regression Analysis for OS.

Variable	HR (95% CI)	*p*-Value
Age (continuous)	1.015 (1.001–1.030)	0.038
CRP	1.000 (0.998–1.002)	0.977
Albumin	1.046 (0.813–1.346)	0.727
CA 19-9	1.048 (0.992–1.108)	0.095
CEA	1.188 (1.088–1.298)	<0.001
First-Line Chemotherapy (FX vs. Gem)	0.987 (0.704–1.384)	0.940
Liver Metastasis	1.375 (0.976–1.935)	0.068
Intra-abdominal LN Metastasis	1.380 (1.051–1.812)	0.021
Bone Metastasis	1.818 (1.379–2.397)	<0.001
EASIX Class (High vs. Low)	4.094 (1.993–8.411)	<0.001

Abbreviations: OS, overall survival; HR, hazard ratio; CI, confidence interval; EASIX, Endothelial Activation and Stress Index; CA 19-9, carbohydrate antigen 19-9; CEA, carcinoembryonic antigen; LN, lymph node; FX, FOLFIRINOX (5-fluorouracil, leucovorin, irinotecan, oxaliplatin); CRP, C-reactive protein.

## Data Availability

The datasets generated and/or analyzed during the current study are available from the corresponding author on reasonable request. The data are not publicly available due to ethical restrictions and patient confidentiality.
